# Perspectives, fears and expectations of patients with gynaecological cancers during the COVID‐19 pandemic: A Pan‐European study of the European Network of Gynaecological Cancer Advocacy Groups (ENGAGe)

**DOI:** 10.1002/cam4.3605

**Published:** 2020-11-18

**Authors:** Murat Gultekin, Sertac Ak, Ali Ayhan, Aleksandra Strojna, Andrei Pletnev, Anna Fagotti, Anna Myriam Perrone, B. Emre Erzeneoglu, B. Esat Temiz, Birthe Lemley, Burcu Soyak, Cathy Hughes, David Cibula, Dimitrios Haidopoulos, Donal Brennan, Edoardo Cola, Elzbieta van der Steen‐Banasik, Esra Urkmez, Huseyin Akilli, Ignacio Zapardiel, Icó Tóth, Jalid Sehouli, Kamil Zalewski, Kiarash Bahremand, Luis Chiva, Mansoor Raza Mirza, Maria Papageorgiou, Novak Zoltan, Petra Adámková, Philippe Morice, Sonia Garrido‐Mallach, Utku Akgor, Vasilis Theodoulidis, Zafer Arik, Karina D. Steffensen, Christina Fotopoulou

**Affiliations:** ^1^ Faculty of Medicine Department of Obstetrics and Gynecology Division of Gynaecological Oncology Hacettepe University Ankara Turkey; ^2^ European Society of Gynaecological Oncoloy (ESGO European Network of Gynaecological Cancers Advocacy Groups (ENGAGe) Executive Group Prague Czech Republic; ^3^ Stress Assesment and Research Center (STAR Hacettepe University Ankara Turkey; ^4^ Faculty of Medicine Department of Psychiatry Hacettepe University Ankara Turkey; ^5^ Faculty of Medicine Department of Obstetrics and Gynecology Division of Gynaecological Oncology Baskent University Ankara Turkey; ^6^ Department of Gynecological Surgery and Gynecological Oncology of Adults and Adolescents Pomeranian Medical University Szczecin Poland; ^7^ Department of Gynecologic Oncology N.N. Alexandrov National Cancer Center of Belarus Minsk Belarus; ^8^ Fondazione Policlinico Universitario A. Gemelli IRCCS Catholic University of the Sacred Heart Rome Italy; ^9^ Sant Orsola‐Malpighi Hospital Bologna Italy; ^10^ KIU – Patient Organisation for Women with Gynaecological Cancer Copenhagen Denmark; ^11^ Ovacome ‐ Ovarian Cancer Support Charity London UK; ^12^ Department of Gynecology Imperial College London NHS Trust London London UK; ^13^ Department of Obstetrics and Gynecology Gynecologic Oncology Center First Faculty of Medicine Charles University and General University Hospital Prague Czech Republic; ^14^ Department of Obstetrics and Gynecology Alexandra Hospital University of Athens Athens Greece; ^15^ Department of Gynaecological Oncology University College Dublin (UCD) School of Medicine Catherine McAuley Research Centre Mater University Hospital Dublin Ireland; ^16^ Radiotherapy Institute Arnhem The Netherlands; ^17^ Dance With Cancer Society Ankara Turkey; ^18^ Gynecologic Oncology Unit La Paz University Hospital‐IdiPAZ Madrid Spain; ^19^ Mallow Flower Foundation Budapest Hungary; ^20^ Department of Gynecology with Center for Oncological Surgery Charité‐University Hospital Berlin Germany; ^21^ Department of Gynecological Oncology Hollycross Cancer Center Kielce Poland; ^22^ Department of Molecular and Translational Oncology Maria Sklodowska‐Curie National Research Institute of Oncology Warsaw Poland; ^23^ Department of Gynaecological Oncology Barts Health NHS Trust London UK; ^24^ Department of Gynaecology National Institute of Oncology Budapest Hungary; ^25^ Clinica Universidad de Navarra Madrid Spain; ^26^ Department of Oncology The Finsen Centre Rigshospitalet ‐ Copenhagen University Hospital Copenhagen Denmark; ^27^ K.E.F.I. Cancer Society Athens Greece; ^28^ Onko Unie Cancer Society Prague Czech Republic; ^29^ Department of Surgery Institute Gustave Roussy Villejuif France; ^30^ Faculty of Medicine Department of Medical Oncology Hacettepe University Ankara Turkey; ^31^ Department of Oncology Lillebaelt Hospital‐University Hospital of Southern Denmark Vejle Denmark; ^32^ Department of Regional Health Research Faculty of Health Sciences University of Southern Denmark Odense Denmark; ^33^ Center for Shared Decision Making Lillebaelt Hospital‐University Hospital of Southern Denmark Vejle Denmark

**Keywords:** anxiety, cancer, COVID‐19, depression, EU, expectation, gynaecologic oncology, patients, perspectives

## Abstract

**Background:**

The impact of the COVID‐19 pandemic on European gynaecological cancer patients under active treatment or follow‐up has not been documented. We sought to capture the patient perceptions of the COVID‐19 implications and the worldwide imposed treatment modifications.

**Methods:**

A patient survey was conducted in 16 European countries, using a new COVID‐19‐related questionnaire, developed by ENGAGe and the Hospital Anxiety & Depression Scale questionnaire (HADS). The survey was promoted by national patient advocacy groups and charitable organisations.

**Findings:**

We collected 1388 forms; 592 online and 796 hard‐copy (May, 2020). We excluded 137 due to missing data. Median patients’ age was 55 years (range: 18–89), 54.7% had ovarian cancer and 15.5% were preoperative. Even though 73.2% of patients named cancer as a risk factor for COVID‐19, only 17.5% were more afraid of COVID‐19 than their cancer condition, with advanced age (>70 years) as the only significant risk factor for that. Overall, 71% were concerned about cancer progression if their treatment/follow‐up was cancelled/postponed. Most patients (64%) had their care continued as planned, but 72.3% (*n* = 892) said that they received no information around overall COVID‐19 infection rates of patients and staff, testing or measures taken in their treating hospital. Mean HADS Anxiety and Depression Scores were 8.8 (range: 5.3–12) and 8.1 (range: 3.8–13.4), respectively. Multivariate analysis identified high HADS‐depression scores, having experienced modifications of care due to the pandemic and concern about not being able to visit their doctor as independent predictors of patients’ anxiety.

**Interpretation:**

Gynaecological cancer patients expressed significant anxiety about progression of their disease due to modifications of care related to the COVID‐19 pandemic and wished to pursue their treatment as planned despite the associated risks. Healthcare professionals should take this into consideration when making decisions that impact patients care in times of crisis and to develop initiatives to improve patients’ communication and education.

## Graphical abstract

Gynaecological cancer patients in Europe appeal for more transparency and continuation of high standard oncologic care even in times of crisis like the COVID‐19 pandemic.

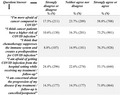

## 1 INTRODUCTION

Our society, and the world as we know it, has been transformed in unprecedented ways by the COVID‐19 pandemic. Healthcare systems have been challenged on multiple levels across all continents and modifications of care were urgently undertaken in order to adjust to a dynamic rapidly evolving public health emergency.^1^ As demands on physical infrastructure and personnel resources were pushed to their limits, the weakest many vulnerable patient groups, such as those with advanced and chronic cancer conditions, have been most affected.^1^, ^2^, ^3^, ^4^, ^5^ The strict rules, introduced by many governments, created priority levels to which patients treatment would have to be modified and in some cases postponed, to facilitate the rapidly developing healthcare restrains and to increase the availability for patients with COVID‐19.^6^, ^7^, ^8^, ^9^, ^10^, ^11^, ^12^, ^13^ Overall the considerable higher demands for intensive care monitoring and ventilation of cancer versus non‐cancer patients, led a common fear among clinicians and regulators about that many healthcare systems would be overrun.^14^, ^15^, ^16^

Moreover, discouraging experiences from China^14^, ^15^ and additional international studies,^16^ demonstrated a significant increase in COVID‐19‐related morbidity and mortality in cancer patients under active treatment. Surgical mortality in cancer patients who develop peri‐operative COVID‐19 has been reported to be as high as 25%,^14^, ^15^, ^16^ which led to the postponement and modification of surgical and chemotherapy protocols during the peak of the pandemic.

Gynaecological cancers represent a major part of the total cancer burden in women.^17^ It is estimated that each year half a million deaths are caused worldwide by ovarian‐, cervical‐, endometrial and vulva‐/ vaginal cancers with an incidence of over one million new cases.^18^ All major gynae‐oncology societies released consistent recommendations to minimise the staff, family and patient exposure to the virus at the onset of the pandemic. Significant modifications of the main treatment pathways such as postponing elective surgeries, increasing rates of neo‐adjuvant chemotherapy in advanced disease, reducing/ stopping surgery at relapse and postponing routine follow‐up/surveillance visits with transition to telemedicine/web‐based consultation were also introduced.^6^, ^7^, ^8^, ^9^, ^10^, ^11^, ^12^, ^13^

The urgency of the situation required dynamic decision‐making processes across all levels, with little or no time to incorporate or even consider any patients perspectives. However, increasing cancer deaths are likely to be a major outcome of this pandemic and it is critical that the patients voice is expressed and presented in a public forum, so that we can learn from this episode and plan for future waves of COVID‐19 or similar crisis.^19^ Under this perspective, the European Network of Gynaecological Cancer Advocacy Groups (ENGAGe), a network established by ESGO, conducted a survey among gynaecological cancer patients across Europe to capture how women with gynaecological cancers perceived the modifications of care and impact of the pandemic on their personal journey.

## 2 METHODS

### 2.1 Patients and set up

This is a prospective survey study conducted in 16 European countries (France, United Kingdom [UK], Italy, Spain, Greece, Turkey, Czech Republic, Germany, Netherlands, Denmark, Poland, Serbia, Hungary, Belarus, Ireland and Finland), online and on paper, between 1st and 30th of May 2020. All patients above 18 years of age with gynaecological cancers of any stage, histology and type were eligible to participate as long as they were still under active treatment or surveillance. Depending on the stage of their treatment journey, patients were divided into three categories: type 1 with a diagnosis of primary or recurrent cancer scheduled for surgery; type 2 when receiving chemotherapy and/or radiotherapy for primary or recurrent disease (neoadjuvant chemotherapy and maintenance targeted treatment was included here) and type 3 when under routine oncologic follow‐up. Patients under palliative care alone at home or in the community were not included into this study. Since we evaluated anxiety and depression scales, we excluded all patients who had a previous diagnosis of a psychiatric disorder, unrelated to their cancer diagnosis, that required medication such as bipolar disorder or schizophrenia. Ethical committee approval of the study was obtained from the Hacettepe University (16969557‐580) in Turkey and a consortium protocol was sent to all participating countries for local ethical approval as per each country's regulations. When the study was promoted online via social media from patients Charities, no additional ethical approval was required. The survey was completely anonymous and no personal identification information in any form like name, initials or date of birth was requested or recorded.

### 2.2 Development of the survey

The survey study consisted of two parts: one COVID‐19 related (sections A and B) and the well‐established and validated 14‐item Hospital Anxiety and Depression Scale (HADS).^20^, ^21^ Sections A and B were developed by the investigators in the Hacettepe University including gynaecological and medical oncologists and psychiatrists. A full copy of the survey questionnaire in English is found in the supplement of this article. In order to apply the survey in different European countries, sections A and B were translated by local ESGO clinicians and ENGAGe members. A validated HADS form is available in 115 languages and, therefore, suitable for researchers internationally. The use of the HADS questionnaire is licenced by GL Assessment Ltd., Swindon, UK. A licence agreement was completed and a user fee was paid for the validated translations required for the present study. The HADS is a self‐report rating scale of 14 items on a 4‐point Likert scale (range 0–3). It is designed to measure anxiety and depression (seven items for each subscale). Some questions determine anxiety while the others determine depression. HADS‐questionnaires have a maximum score of 21. Scores of ≥11 on either subscale are considered to be a significant ‘case’ of psychological morbidity (abnormal), while scores of 8–10 represents ‘borderline’ and 0–7 ‘normal’ (healthy individuals).

To ensure applicability, the initially developed draft was circulated among the ENGAGE executive committee that included three clinicians and five gynaecological cancer survivors from different European countries for their feedback. The survey was then sent to all national ENGAGe member organisations (>40) for final feedback and comments, to ensure Pan‐European patient involvement in the development and applicability of the COVID‐19‐related questionnaire. ENGAGe organisations were specifically asked to assess the intelligibility and comprehensibility of the questions in local lay language. Once the survey was finalised, it was uploaded to the internet as an online questionnaire (Survey Monkey) and, therefore, both online and hard copy surveys were available to the participants. The final version of the survey is presented in Data S1 of this article.

### 2.3 Distribution and promotion of the survey

At an executive board level, ENGAGe and the investigators ensured that no unnecessary or additional hospital or clinic visits occurred just for the purposes of the survey. Depending on the local needs, customs and system modifications of each country, surveys were distributed to the patients via different channels either through the treating clinical team or via the national patient charities and advocacy groups. Individual patients also promoted the survey link via social media platforms and patient forums. In the countries where the online survey was not available in the local language, the online survey link in English was uploaded together with a translated document file, so that patients had access also to the translated version via the social media channels of the local ENGAGe organisations. ENGAGe members additionally organised online live broadcast tele‐conferences for their members to explain and assist in any questions regarding the online survey (2.9% of the whole study population). All these measures were undertaken to overcome any barriers induced by social isolation, language or technical difficulties especially faced by elderly patients.

### 2.4 Statistical analysis

The online survey data were automatically collected from the server. Hard copy survey data were entered by the local study investigators into excel or SPPS forms and sent centrally for analysis. Questionnaires with two or more missing or invalid question items were excluded from the study.

Multivariate logistic regression analysis was used to determine predictors of ‘having severe anxiety’, ‘having severe depression’ and ‘expressing more fear from COVID‐19 compared to cancer’. These dependent variables were coded as categorical variables (0 and 1). The independent variables that took place in the Regression model were established as ordinal. All of the Likert‐type questions were categorised into two; according to the study aims strongly disagree, disagree and nor agree or disagree were coded as ‘0’; while the agree and strongly agree were coded as ‘1’. For all variables in the equation Odds ratios and lower and upper levels of 95% of Confidence intervals were calculated and the Odds ratios assumed as statistically meaningful *p* values’ lower than 0.05. All data were collected and evaluated with Microsoft Excel and SPSS version 25.0. (IBM Corp.).

## 3 RESULTS

### 3.1 Patients population

A total of 1388 survey forms were collected from 1st May 2020 to 31st May 2020; 592 were completed online and 796 on hard copy. Of those, 137 were excluded due to missing data in ≥2 question items. No patients were excluded due to a concomitant unrelated psychiatric disorder. A total of 1251 questionnaires were included in the final analysis. We did not collect the number of patients who have refused the online or hard copy surveys.

Median patients age was 55 years (range: 18–89). Only 32 women were younger than 30 years (2.6%) and 141 (11.3%) belonged to the elderly group of 70 years of age or older. COVID‐19 measures were strictly applied for elderly patient population globally (>70 years) and the risk of COVID‐19‐associated mortality is exponentially increasing in patients over 70 years compared to younger ones. For that reason, we wished to evaluate whether this had any impact on the patient's perception and anxiety levels. Furthermore, many gynaecological oncology papers use the cut off of 70 years to define surgical morbidity in elderly patients versus the younger ones.^22^

The highest number of patients came from Hungary (*n* = 165; 13.2%), while 7 of the participating countries recruited at least 100 patients or more, so that we ensured geographical balance within Europe. The majority of the patients (*n* = 627; 54.8%) had ovarian cancer, 224 (19.6%) uterine/ endometrial cancer, 198 (17.3%) cervical cancer and the remaining 96 (8.4%) patients had other rarer types. Regarding stage of their treatment journey, 185 (15.4%) were preoperative (Type‐1); 553 (46.1%) patients reported receiving chemotherapy or radiotherapy for primary or recurrent disease (Type‐2) and 463 (35.6%) patients were on follow‐up surveillance programmes (Type‐3). A total of 554 patients (44.3%) reported having at least one co‐morbidity. Of these, 182 (14.8%) patients had two; and 100 (8.1%) three or more co‐morbid conditions. Only 134 (10.7%) of the patients reported current use of anti‐depressant medication. Demographics of the study population are presented in Table 1.

**TABLE 1:** Demographics and HADS scores of patients per country of origin. (Type 1: Preoperative patients––surgery is planned for a new diagnosis of cancer or recently recurred cancer; Type 2: Receiving chemotherapy or radiotherapy, before or after surgery, for primary or recurrent disease); Type 3: Not receiving any treatment, only on follow‐up).

	Belarus (%)	Czech Republic (%)	Denmark (%)	France (%)	Finland (%)	Germany (%)	Greece (%)	Hungary (%)	Ireland (%)	Italy (%)	Netherlands (%)	Poland (%)	Serbia (%)	Spain (%)	Turkey (%)	UK (%)	Other (%)	Total (%)
Age																		
<30 Years	1 (0·1)	2 (0·2)	0 (0)	0 (0)	0 (0)	2 (0·2)	2 (0·2)	5 (0·4)	0 (0)	7 (0·6)	0 (0)	1 (0·1)	0 (0)	4 (0·3)	4 (0·3)	4 (0·3)	0 (0)	32 (2·6)
30–70 Years	56 (4·5)	11 (0·9)	31 (2·5)	24 (1·9)	14 (1·1)	39 (3·1)	108 (8·6)	149 (11·9)	40 (3·2)	140 (11·2)	35 (2·8)	121 (9·7)	12 (1)	89 (7·1)	105 (8·4)	79 (76·3)	25 (2)	1078 (86·2)
>70 Years	2 (0·2)	0 (0)	16 (0·5)	2 (0·2)	0 (0)	1 (0·1)	23 (1·8)	11 (0·9)	2 (0·2)	14 (1·1)	4 (0·3)	20 (1·6)	0 (0)	16 (1·3)	24 (1·9)	10 (0·8)	6 (0·5)	141 (11·3)
Total	59 (4·7)	13 (1)	37 (3)	26 (2·1)	14 (1·1)	42 (3·4)	133 (10·6)	165 (13·2)	42 (3·4)	161 (12·9)	39 (3·1)	142 (11·4)	12 (1)	109 (8·7)	133 (10·6)	93 (7·4)	31 (2·5)	1251 (100)
Diagnosis																		
Ovarian	14 (1·1)	9 (0·7)	24 (1·9)	16 (1·3)	8 (0·6)	31 (2·5)	61 (4·8)	63 (5)	19 (1·5)	110 (69·1)	19 (1·5)	60 (4·8)	0 (0)	36 (2·9)	71 (5·6)	70 (5·5)	16 (1·3)	627 (50·1)
Uterine	11 (0·9)	0 (0)	0 (0)	4 (0·3)	2 (0·2)	0 (0)	35 (2·8)	13 (1)	5 (0·4)	28 (17·6)	5 (0·4)	50 (3·9)	0 (0)	44 (3·5)	20 (1·6)	4 (0·3)	3 (0·3)	224 (17·9)
Cervical	27 (2·1)	1 (0·1)	3 (0·3)	2 (0·2)	2 (0·2)	3 (0·3)	23 (1·8)	47 (3·7)	4 (0·3)	18 (11·3)	8 (0·6)	21 (1·7)	1 (0·1)	24 (1·9)	9 (0·7)	2 (0·2)	3 (0·3)	198 (15·8)
Others	5 (0·4)	3 (0·3)	1 (0·1)	4 (0·3)	2 (0·2)	2 (0·2)	11 (0·9)	4 (3·2)	4 (0·3)	3 (2)	2 (0·2)	7 (0·6)	10 (0·8)	5 (0·4)	12 (1)	13 (1)	8 (0·6)	96 (7·6)
Missing	2 (0·2)	0 (0)	9 (0·7)	0 (0·	0 (0)	6 (0·5)	3 (0·3)	38 (3)	10 (0·8)	2 (0.2)	5 (0·4)	4 (0·3)	1 (0·1)	0 (0)	21 (1·7)	4 (0·3)	1 (0·1)	106 (8·4)
Total	59 (4·7)	13 (1)	37 (3)	26 (2·1)	14 (1·1)	42 (3·4)	133 (10·6)	165 (13·2)	42 (3·4)	161 (12·9)	39 (3·1)	142 (11·4)	12 (1)	109 (8·7)	133 (10·6)	93 (7·4)	31 (2·5)	1251 (100)
Treatment																		
Type‐1	25 (2)	1 (0·1)	0 (0)	7 (0·6)	0 (0)	6 (0·5)	45 (3·6)	12 (1)	3 (0·3)	37 (2·9)	3 (0·3)	17 (1·3)	0 (0)	13 (1)	6 (0·5)	6 (0·5)	4 (0·3)	185 (14·7)
Type‐2	15 (1·2)	6 (0·5)	17 (1·3)	13 (1)	5 (0·4)	23 (1·8)	50 (4)	61 (4·9)	11 (0·9)	89 (7·1)	21 (1·7)	32 (2·5)	10 (0·8)	33 (2·6)	107 (8·5)	43 (3·4)	17 (1·3)	553 (44·2)
Type‐3	3 (0·3)	5 (0·4)	18 (1·4)	6 (0·5)	8 (0·6)	13 (1)	38 (3)	85 (6·8)	28 (2·2)	35 (2·8)	13 (1)	91 (7·2)	2 (0·2)	63 (5)	5 (0·4)	40 (3·2)	10 (0·8)	463 (38·5)
Missing	16 (1·3)	1 (0·1)	2 (0·2)	0 (0)	1 (0·1)	0 (0)	0 (0)	7 (0·6)	0 (0)	0 (0)	2 (0·2)	2 (0·2)	0 (0)	0 (0)	15 (1·2)	4 (0·3)	0 (0)	50 (3·9)
Total	59 (4·7)	13 (1)	37 (3)	26 (2·1)	14 (1·1)	42 (3·4)	133 (10·6)	165 (13·2)	42 (3·4)	161 (12·8)	39 (3·1)	142 (11·4))	12 (1)	109 (8·7)	133 (10·6)	93 (7·4)	31 (2·4)	1251 (100)
HADS Depression (Mean Score)	5·7	8·2	3·8	6·4	7·4	5·9	6·6	5·5	6·3	11·5	5·3	13·4	7·5	6·8	6 (0·5)	7·2	8·3	8·1
HADS Anxiety (Mean Score)	6·9	9·9	5·3	7·6	8·3	8·3	7·9	7·5	7·4	10·8	6·6	12·0	8·9	8·3	9·2	9·4	8·6	8·8

### 3.2 COVID‐19‐related analysis and patients’ views

Even though the vast majority of women (*n* = 901; 73.2%) thought that cancer patients were at higher risk of a COVID‐19 infection mainly due to chemotherapy‐induced suppression of their immune system; only a minority of them (*n* = 211; 17.5%) were actually more afraid of COVID‐19 than their pre‐existing malignant diagnosis. Most patients (*n* = 864; 71%) were concerned that their cancer would progress as a result of delay or cancellation of their treatment or oncologic follow‐up. Approximately half of the patients (53.1%) expressed their fear of contracting COVID‐19 from the hospital or clinic while receiving their oncologic treatment or follow‐up (Table 2).

**TABLE 2:** COVID‐19‐related fears of patients with gynaecological cancers during the COVID‐19 pandemic.

Question/answer	Strongly disagree or disagree % (N)	Neither agree nor disagree % (N)	Strongly agree or agree % (N)
‘I'm more afraid of cancer compared to COVID’	17.5% (211)	23.7% (289)	58.8% (708)
‘I think cancer patients have a higher risk of COVID infection’	10.6% (130)	16.3% (201)	73.2% (901)
‘I think that chemotherapy suppresses the immune system and creates a predisposition for COVID infection’	8.8% (107)	14.9% (181)	76.3% (928)
‘I am afraid of getting COVID infection from the hospital setting while receiving my treatment/follow‐up’	24.4% (296)	22.6% (274)	53.1% (644)
‘I am concerned about the progression of my disease if my treatment/follow‐up is cancelled/postponed’	14.5% (177)	14.5% (177)	71.0% (864)

In the multivariate, regression analysis, advanced age of 70 years or older was the only risk factor for ‘being more afraid of COVID‐19 compared to cancer’; whereas other factors such as being on active treatment or just surveillance, having additional comorbidities, having metastatic disseminated disease such as ovarian cancer, knowing that other COVID‐19‐infected individuals (doctors or patients) were present in the treating hospital and having experienced modification of care due to the pandemic, did not have any significant effect on the patients fear of the pandemic over cancer (Table 3).

**TABLE 3:** Risk factors for more ‘being more afraid of COVID compared to cancer’: multivariate analysis (logistic regression).

Variable	Odds ratio	95% Confidence interval		*p* value
		Lower	Upper	
Age (≥70 vs. <70 years)	4.09	2.01	8.32	<0.001
Type of treatment (1 or 2 vs. 3)	0.68	0.38	1.21	0.19
Ovarian cancer (yes vs. no)	1.12	0.60	1.90	0.68
Additional comorbidities (yes vs. no)	1.53	0.91	2.58	0.11
Experienced modification of care due to the pandemic (of any type) (yes vs. no)	1.29	0.74	2.24	0.37
Presence of COVID‐19 infected individuals (patients or doctors) in the hospital where the patient is treated (yes vs. no)	0.8	0.44	1.45	0.45

In terms of impact of the COVID‐19 pandemic on patients care; 64% (*n* = 772) stated that their care continued as previously planned despite the pandemic, while only 89 (7.4%) patients reported not attending their treatment/ follow‐up appointments due to fear of COVID‐19 infection. Also, a rather small minority (*n* = 156; 12.9%) stated that they wanted to go themselves, but their doctors cancelled their appointments and 96 (7.9%) patients said that the postponement of their planned treatment was a joint decision between themselves and their treating team.

More than half of the participating patients (*n* = 699; 56.5%) stated that they were not aware if any other COVID‐19 affected patients were treated in the hospital/clinic where they also received treatment. Only 21.4% (*n* = 263) of the patients had COVID‐19 testing before or during their treatment. As expected, only 2.4% of the participating patients reported that the healthcare professionals providing them medical treatment had a COVID‐19 infection and 72.3% (*n* = 892) said that they had no information regarding that at all.

Of the 390 (32.6%) patients that reported their treatment or follow‐up having been changed and/or modified due to the pandemic; 77 (6.2%) expressed difficulty reaching their doctor, only 64 (5.1%) said that their surgery was delayed, 87 (7%) said that their imaging was cancelled or disrupted, 35 (2.8%) reported a delay in their chemotherapy or radiotherapy (6; 0.5%) appointments, while 160 (12.8%) reported that their follow‐up was postponed or delayed. Upon the question whether and how many weeks their oncological care was postponed; 668 (53.4%) patients declined any postponement or cancellation, 132 (10.5%) patients responded that there was a delay and that they would not know how long and 135 (10.8%) patients gave a delay of their treatment (median: 6.2 weeks). In regards to clinical trial participation, 114 (9.1%) of the patients stated receiving a drug as part of a clinical research programme and only four of those patients said that the trial was stopped and that they did not have any further access to the tested drug. Almost all of the patients who were part of a clinical trial (110/114) expressed their wish to continue clinical trial participation even during the pandemic.

There were two open‐ended questions in this part of the survey. The first one ‘what is the most challenging problem in this period?’ was answered by 623 patients. Two hundred and seventy‐four patients (44%) expressed their concerns related to the uncertainty that the pandemic has created, while only 13 patients (2.0%) named financial aspects induced by the pandemic as a challenging problem.

The second open‐ended question ‘Message that you want told to share about COVID‐19 pandemic with ESGO, ENGAGe and Other International Organizations’ was answered by 156 women. Ninety‐nine patients (65%) spoke about ‘cancer being more lethal than Covid‐19’ and that something must be done to protect cancer patients, naming as examples special products or measures and COVID‐19‐free cancer hospitals. Twenty‐two patients (14%) used this as an opportunity to express their thanks and gratitude to their doctors and healthcare societies.

### 3.3 HADS Anxiety and Depression Scores

**FIGURE 1:**
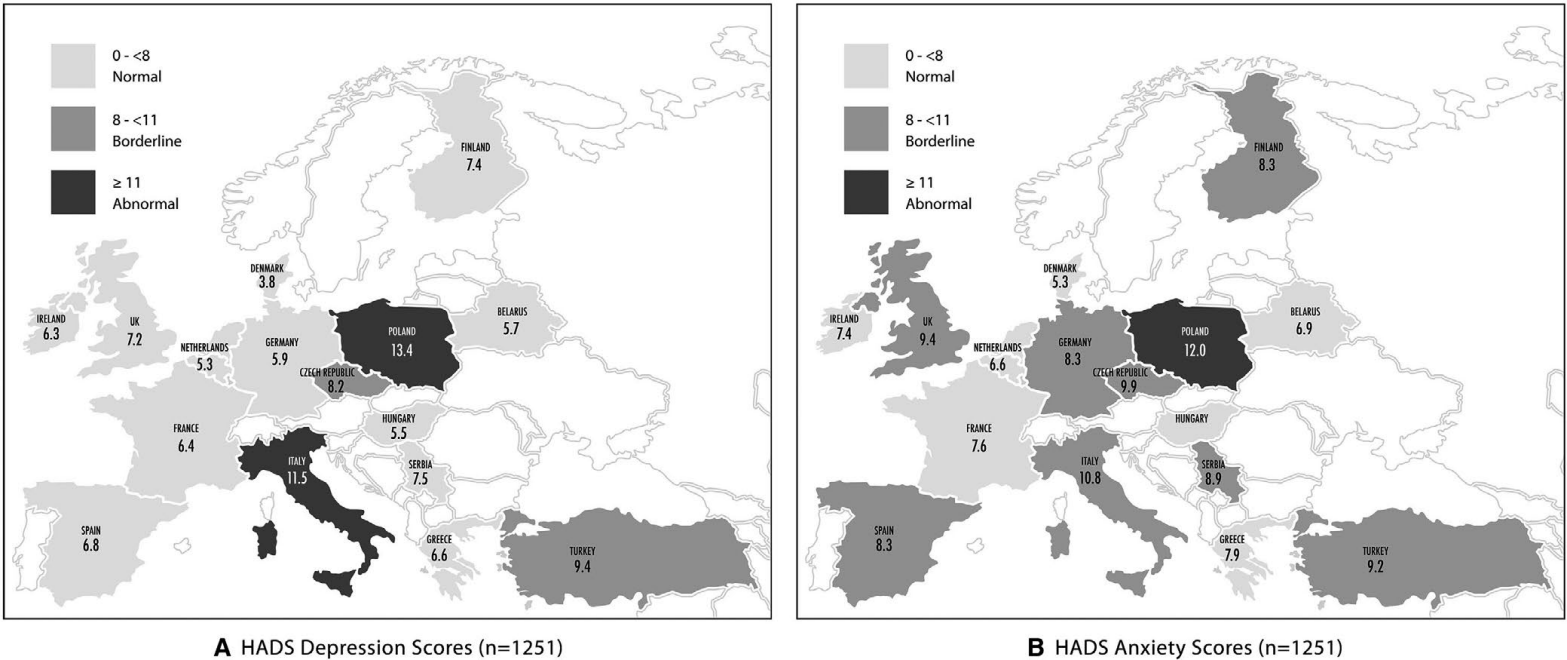
HADS Depression (A) and Anxiety (B) Scores

Mean HADS Anxiety Score (HADS‐A) was 8.8 (range: 5.3–12) and mean HADS Depression Score (HADS‐D) was 8.1 (range: 3.8–13.4). Detailed HADS anxiety and depression scores per country are represented in Table 1 and Figure 1.

Five hundred and eight (40.6%) women had a normal, 301 (24.1%) had a borderline and 442 (35.3%) had an abnormal HADS Anxiety score; whereas the equivalent HADS Depression score was: 610 (48.8%), 258 (20.6%) and 383 (30.6%), respectively.

In the multivariate logistic regression analysis, we could identify a HADS Depression score of ≥11 (OR: 11.98; 95% CI: 8.52–16.84), having experienced modification of care due to the pandemic of any type (OR: 1.52; 95% CI: 1.07–2.1) and concerned about not being able to visit the oncology doctor during the COVID‐19 pandemic (OR: 1.94; 95% CI: 1.34–2.8) as being associated with a significantly higher risk for an abnormal (i.e. 11–21) HADS Anxiety score. Age, type of treatment or cancer, additional comorbidities, being afraid more of COVID‐19 than fear or being concerned about the progression of cancer if treatment/follow‐up were cancelled or postponed, did not have any significant effect on patients’ anxiety levels (Table 4).

**TABLE 4:** Risk factors for abnormal (i.e. 11–21) HADS anxiety score: multivariate analysis (logistic regression).

Variable	Odds ratio	95%Confidence interval		*p* value
		Lower	Upper	
Age (≥70 vs. <70 years)	1.24	0.74	2.08	0.41
Type of treatment (1 or 2 vs. 3)	0.8	0.57	1.13	0.20
HADS depression score (≥11 vs. <11)	11.98	8.52	16.84	<0.001
Additional comorbidities (yes vs. no)	1.27	0.91	1.76	0.16
Experienced modification of care due to the pandemic (of any type) (yes vs. no)	1.52	1.07	2.16	0.02
COVID‐19 fear more than cancer fear (yes vs. no)	1.04	0.7	1.54	0.86
Ovarian cancer (yes vs. no)	1.08	0.78	1.49	0.66
Concerned about not being able to visit the oncology doctor during the COVID‐19 pandemic (yes vs. no)	1.94	1.35	2.80	<0.001
Concerned about the progression of cancer if treatment/follow‐up is cancelled/postponed (yes vs. no)	1.05	0.70	1.56	0.82

For patients presenting with abnormal HADS Depression scores (ie ≥11), multivariate analysis did not identify age, type of treatment or cancer, having experienced modifications of care due to the pandemic, being afraid more of COVID‐19 than cancer and being concerned about the progression of cancer if treatment/follow‐up was cancelled or postponed as independent prognostic factors, but solely abnormal HADS Anxiety scores (OR: 12.02; 95% CI: 8.55–16.9) and presence of additional comorbidities (OR: 1.522; 95% CI: 1.08–2.13). Patients who were concerned about not being able to visit their oncology doctor during the COVID‐19 pandemic were significantly less likely to show high depression scores (OR: 0.652; 95% CI: 0.449–0.949) (Table 5).

**TABLE 5:** Risk factors for abnormal (i.e. 11–21) HADS depression score: multivariate analysis (logistic regression).

Variable	Odds ratio	95% Confidence interval		*p* value
		lower	upper	
Age (≥70 vs. <70 years)	0.84	0.49	1.42	0.51
Type of treatment (1 or 2 vs. 3)	0.86	0.6	1.22	0.39
HADS anxiety score (≥11 vs. <11)	12.02	8.55	16.9	<0.001
Additional comorbidities (yes vs. no)	1.52	1.09	2.13	0.02
Experienced modification of care due to the pandemic (of any type) (yes vs. no)	0.75	0.52	1.08	0.12
COVID‐19 fear more than cancer fear (yes vs. no)	1.15	0.77	1.71	0.51
Ovarian cancer (yes vs. no)	0.88	0.63	1.22	0.44
Concerned about not being able to visit the oncology doctor during the COVID‐19 pandemic (yes vs. no)	9.65	0.45	0.95	0.03
Concerned about the progression of cancer if treatment/follow‐up is cancelled/postponed (yes vs. no)	1.24	0.83	1.86	0.30

## 4 DISCUSSION

We present the findings of the largest survey, to the best of our knowledge that captures the views, fears and perspectives of patients with gynaecological cancer in Europe related to the impact and modifications of their care due to the COVID‐19 pandemic. With 1251 responders from 16 European countries we achieved a large geographical coverage in an effort to overcome any local bias. Also, by having both online and paper questionnaires, we wished to eliminate the known biases of online surveys and distribution processes. We demonstrated that even though, as expected, most patients named cancer as a major risk factor for developing COVID‐19, less than one fifth of them were actually more afraid of COVID‐19 than their cancer condition and this was mostly the case among the elderly patients over 70 years of age. Although patients were aware of their increased risk of developing COVID‐19, their main concern was still the potential to develop progressive disease as a result of treatment disruption during the pandemic. For that reason, more than 90% of patients tried to attend their planned treatment appointments as originally scheduled.

As anticipated, feelings of anxiety and depression were strongly cross‐correlated. The inability to visit their treating team as well as experiencing modifications of care due to the pandemic, significantly contributed to high patients’ anxiety levels. With growing body of evidence that psychological distress might have some predictive capacity for cancer presentation and progression,^23^ it is even more important that we as clinicians do not additionally feed into patients’ stress through our actions and to carefully balance any measures we plan in order to adapt to newly emerging situations. Conversely, women with high depression scores appeared as if they were resigned to the situation and were significantly less concerned whether they could reach or visit their doctor. Interestingly, ‘being more afraid of COVID‐19 than cancer’ did not significantly affect anxiety or depression levels.

The clear wish of the patient to continue their participation in clinical trials, something associated with potentially more physical hospital visits and increased morbidity, demonstrates their desire to proceed with maximum therapeutic effort even they are most vulnerable.

The hot‐spots of the COVID‐19 pandemic in Europe were, as expected, reflected on the HADS anxiety and depression scores we registered. United Kingdom, Italy, France and Spain which have been the most affected countries in Europe registering 27,000–40,000 COVID‐19‐related deaths,^24^ indeed showed abnormal HADS scores with Italy leading probably due to the fact that it was the first heavily affected European country. However, also other areas such as Turkey and Eastern Europe recorded some of the highest anxiety and depression levels in our survey even though the number of new cases and deaths from the COVID‐19 pandemic were some of the lowest in Europe.^24^ This might potentially indicate that it is not just the fear for the virus itself that causes patients distress, but rather the implications that the pandemic has on the society, with some countries being able to possibly cope and adapt more readily depending on their resources and mechanisms of restructuring care.

Surprisingly, cancer type and treatment did not have any significant impact on patients’ anxiety and depression scores. Ovarian cancer was the most common cancer type in our survey, probably due to the chronic condition of the disease with patients being under long‐term treatment and follow‐up, participating in a plethora of clinical trials and often being under maintenance regimens. Given its specific need for extensive cytoreductive surgery and multivisceral resections, in many countries ovarian cancer surgeries had to be postponed or cancelled as a result of the disruption of infrastructural support.^6^, ^7^, ^10^, ^12^ We would have assumed that patients with ovarian cancer would have much higher anxiety and depression levels than those with more favourable cancer types, such endometrial cancer, which however was clearly not the case.

A common theme was throughout a lack of transparency and information flow to the patients regarding the COVID‐19 incidence in their treating hospital and also around COVID‐19 testing, clearly identifying the caveats in how the healthcare system was reshaped to accommodate both COVID‐19 patients and non‐COVID‐19 patients and also staff.

The findings of our study send a clear message to the healthcare community that even in times of crisis, such as natural disasters and pandemics, patients are more concerned about their individual health needs and our responsibility as healthcare professionals is not to fail them despite all challenges. Similar papers in other cancers like breast cancer, show similar findings to us with women with cancers demonstrating significant levels of anxiety, depression and perceived cognitive function due to the COVID‐19 situation. Collectively the authors and oncology community seem to appeal and advocate the rapid implementation of accessible interventions designed to promote emotional resilience of cancer patients in times of crisis.^25^

There are limitations to our study. We almost most certainly failed to reach the older patients with multiple comorbidities, shielding at home alone, too afraid of the pandemic and too scared to reach out to the community. Moreover, hard copies of the survey were completed at hospital appointments during an interaction with their doctors at which they could discuss their concerns and feel reassured. Patients who had relapsed or palliative surgery cancelled and did not receive any systemic treatment in the meantime, would not necessarily had access to the survey due to reduced interaction with their treating teams. Cross‐sectional nature of this survey prevents comparison of present to pre‐pandemic levels of anxiety and depression. We cannot totally exclude the expected baselines increased anxiety and depression scores in cancer patients, despite the fact that analysis is only performed for abnormally high scores. Finally, we were not able to quantify the impact of telemedicine and virtual clinics, many of which were rapidly developed during the pandemic, and may also have provided reassurance to certain subgroups.

Still, our findings provide a solid basis for the healthcare community to improve our understanding of how patients perceive modifications of care in times of crisis and how they set their priorities on a personal level along with an appeal of transparency and information flow. With cancer deaths representing an inevitable collateral damage, patients voice needs to be heard in clinical, regulatory and political decision‐making circles, especially when reinstating care over the upcoming period of the anticipated recovery. These findings would also need to guide future decisions should subsequent waves of COVID‐19 or new pandemics emerge. Even if the governments could not make necessary preventive measures for patients’ anxiety during the pandemic periods, national and international societies can help the patients as much as they can. ESGO family with around 2,500 members have read the message from the cancer patients. Together with ENGAGe, a serial action has been implemented such as COVID‐19 webinars for patients, relaxing and stress reliving teleconferences with patients, an ESGO Task Force for COVID‐19 with the contributions of patients and official COVID‐19 leaflets. These can be implemented globally and patients can be incorporated in to decision‐making analysis during the pandemic periods.

## CONFLICT OF INTEREST

None.

## AUTHOR CONTRIBUTION

**Murat Gultekin:** Conceptualisation, Methodology, Software, Resources, Formal analysis, Investigation, Writing––Original Draft, Writing––Review & Editing, Supervision and Project administration. **Sertac Ak:** Conceptualisation, Methodology, Software, Resources, Data Curation, Formal analysis, Investigation, Writing––Review & Editing, Supervision and Project administration. **Ali Ayhan:** Investigation and Writing––Review & Editing. **Aleksandra Strojna:** Investigation and Writing––Review & Editing. **Andrei Pletnev:** Investigation and Writing––Review & Editing. **Anna Fagotti**: Investigation and Writing––Review & Editing. **Anna Myriam Perrone**: Investigation and Writing––Review & Editing. **B. Emre Erzenoglu:** Software, Investigation and Writing––Review & Editing. **B. Esat Temiz:** Software, Investigation and Writing––Review & Editing. **Birthe Lemley:** Conceptualisation, Methodology, Validation, Investigation, Writing––Review & Editing, Supervision and Project administration. **Burcu Soyak:** Conceptualisation, Methodology, Software, Resources and Formal analysis, **Cathy Hughes:** Investigation and Writing––Review & Editing. **David Cibula:** Investigation and Writing––Review & Editing. **Dimitrios Haidopoulos:** Investigation and Writing––Review & Editing. **Donal Brennan:** Investigation and Writing––Review & Editing. **Edoardo Cola:** Investigation and Writing––Review & Editing. **Elzbieta van der Steen‐Banasik:** Investigation, Writing––Review & Editing. **Esra Urkmez:** Conceptualisation, Methodology, Software, Investigation and Writing––Review & Editing. **Huseyin Akilli:** Investigation and Writing––Review & Editing. **Ignacio Zapardiel;** Investigation and Writing––Review & Editing. **Icó Tóth:**Conceptualisation, Methodology, Validation, Investigation, Writing––Review & Editing, Supervision and Project administration. **Jalid Sehouli:** Conceptualisation, Investigation, and Writing––Review & Editing. **Kamil Zalewski**: Conceptualization, Methodology, Investigation, Writing––Review & Editing, Supervision and Project administration. **Kiarash Bahremand:** Investigation and Writing––Review & Editing. **Luis Chiva:** Investigation and Writing––Review & Editing. **Mansoor Raza Mirza:** Investigation and Writing––Review & Editing. **Maria Papageorgiou:** Conceptualisation, Methodology, Validation, Investigation, Writing––Review & Editing, Supervision and Project administration. **Novak Zoltan:** Investigation and Writing––Review & Editing. **Petra Adámková:** Conceptualisation, Methodology, Validation, Investigation, Writing––Review & Editing, Supervision and Project administration. **Philippe Morice:** Investigation, Writing––Review & Editing, Supervision and Project administration. **Sonia Garrido‐Mallach:** Investigation and Writing––Review & Editing. **Utku Akgor:** Conceptualisation, Methodology, Software, Resources and Formal analysis, **Vasilis Theodoulidis:** Investigation and Writing––Review & Editing. **Zafer Arik:** Investigation and Writing––Review & Editing. **Karina Dahl Steffensen:** Conceptualisation, Methodology, Software, Resources, Investigation, Writing––Review & Editing, Supervision and Project administration. **Christina Fotopoulou:** Conceptualisation, Methodology, Software, Resources, Formal analysis, Investigation, Writing––Original Draft, Writing––Review & Editing, Supervision and Project administration.

## Supporting information

Supplementary Material

## Data Availability

Data available on request from the authors.

## References

[1] Raymond E , Thieblemont C , Alran S , Faivre S . Impact of the COVID‐19 outbreak on the management of patients with cancer. Target Oncol. 2020;22:1‐11. 10.1007/s11523-020-00721-1 PMC7243433 32445083

[2] Glehen O , Kepenekian V , Bouché O , Gladieff L , Honore C , RENAPE‐BIG‐RENAPE . Treatment of primary and metastatic peritoneal tumors in the COVID‐19 pandemic. Proposals for prioritization from the RENAPE and BIG‐RENAPE groups. J Visc Surg. 2020;23:S1878–7886(20), 30118–1. 10.1016/j.jviscsurg.2020.04.013 PMC7177076 32387058

[3] Desideri I , Pilleron S , Battisti NML , et al. Caring for older patients with cancer during the COVID‐19 pandemic: a young international society of geriatric oncology (SIOG) global perspective. Geriatr Oncol. 2020;S1879–4068(20)30215–0. 10.1016/j.jgo.2020.05.001 PMC7252080 32402764

[4] Wang Y , Zhang S , Wei L , et al. Recommendations on management of gynecological malignancies during the COVID‐19 pandemic: perspectives from Chinese gynecological oncologists. J Gynecol Oncol. 2020 10.3802/jgo.2020.31.e68. Online ahead of print PMC7286750 32458596

[5] Vecchione L , Stintzing S , Pentheroudakis G , Douillard JY , Lordick F . ESMO management and treatment adapted recommendations in the COVID‐19 era: colorectal cancer. ESMO Open. 2020;5(Suppl 3):e000826 10.1136/esmoopen-2020-000826 32457036 PMC7276236

[6] https://www.bgcs.org.uk/wp‐content/uploads/2020/05/BGCS‐guidance‐v‐3‐final_‐1.pdf. Accessed November 2020.

[7] https://www.england.nhs.uk/coronavirus/wp‐content/uploads/sites/52/2020/03/specialty‐guide‐acute‐treatment‐cancer‐23‐march‐2020.pdf. Accessed November 2020.

[8] Ramirez PT , Chiva L , Ane Gerda Z , et al. COVID‐19 global pandemic: options for management of gynecologic cancers. Int J Gynecol Cancer. 2020;30(5):561‐563. 10.1136/ijgc-2020-001419. Epub 2020 Mar 27 32221023

[9] https://www.asco.org/asco‐coronavirus‐information/care‐individuals‐cancer‐during‐COVID‐19. Accessed June 2020.

[10] https://www.sgo.org/clinical‐practice/management/COVID‐19‐resources‐for‐health‐care‐practitioners/surgical‐considerations‐for‐gynecologic‐oncologists‐during‐the‐COVID‐19‐pandemic/. Accessed June 2020.

[11] https://www.esmo.org/guidelines/cancer‐patient‐management‐during‐the‐covid‐19‐pandemic. Accessed November 2020.

[12] https://www.esgo.org/esgo‐COVID‐19‐communication/. Accessed November 2020.

[13] COVID‐19Surg Collaborative , Nepogodiev D , Bhangu A . Elective surgery cancellations due to the COVID‐19 pandemic: global predictive modelling to inform surgical recovery plans. Br J Surg, 2020 10.1002/bjs.11746 PMC7272903 32395848

[14] Liang W , Guan W , Chen R , et al. Cancer patients in SARS‐CoV‐2 infection: a nationwide analysis in China. Lancet Oncol. 2020;21(3):335‐337. 32066541 10.1016/S1470-2045(20)30096-6 PMC7159000

[15] Xia Y , Jin R , Zhao J , Li W , Shen H . Risk of COVID‐19 for cancer patients. Lancet Oncol. 2020;21(4):e180 10.1016/S1470-2045(20)30150-9 32142622 PMC7130057

[16] COVID‐19Surg Collaborative . Mortality and pulmonary complications in patients undergoing surgery with perioperative SARS‐CoV‐2 infection: an international cohort study. The Lancet. 2020;396(10243):27‐38. 10.1016/S0140-6736(20)31182-X. PMC7259900 32479829

[17] GCO . Cancer today. http://gco.iarc.fr/today/home. Accessed April 25, 2020.

[18] Sankaranarayanan R , Ferlay J . Worldwide burden of gynecological cancer In: Preedy VR, Watson RR, eds. Handbook of disease burdens and quality of life measures. New York, NY: Springer; 2010:803‐823.

[19] Sud A , Jones M , Broggio J , et al. Collateral damage: the impact on outcomes from cancer surgery of the COVID‐19 pandemic. Ann Oncol. 2020;31(8):1065‐74. 10.1016/j.annonc.2020.05.009 32442581 PMC7237184

[20] Snaith RP . The hospital anxiety and depression scale. Health Qual Life Outcomes. 2003;1:29 10.1186/1477-7525-1-29 12914662 PMC183845

[21] Zigmond AS , Snaith RP . The hospital anxiety and depression scale. Acta Psychiatr Scand. 1983;67(6):361‐370. 10.1111/j.1600-0447.1983.tb09716.x 6880820

[22] Fotopoulou C , Savvatis K , Steinhagen‐Thiessen E , Bahra M , Lichtenegger W , Sehouli J . Primary radical surgery in elderly patients with epithelial ovarian cancer: analysis of surgical outcome and long‐term survival. Int J Gynecol Cancer. 2010;20(1):34‐40. 10.1111/IGC.0b013e3181c10c04. 20130501

[23] David Batty G , Russ Tom C , Emmanuel S , Mika K . Psychological distress in relation to site specific cancer mortality: pooling of unpublished data from 16 prospective cohort studies. BMJ, 2017;356:j108 10.1136/bmj.j108 28122812 PMC5266623

[24] https://covid19.who.int/?gclid=CjwKCAiAtK79BRAIEiwA4OskBlHcoLoJUYbCs9R02bHxNUb9aM5CjkSJ5kMUhdQ_jtiYMhMeHsd2xRoCdM4QAvD_BwE. Accessed November 2020

[25] Swainston J , Chapman B , Grunfeld EA , Derakshan N . COVID‐19 lockdown and its adverse impact on psychological health in breast cancer. Front Psychol. 2020;24(11):2033 10.3389/fpsyg.2020.02033 PMC7476556 32982846

