# Supplementary material for: Perspectives, fears and expectations of patients with gynaecological cancers during the COVID‐19 pandemic: A Pan‐European study of the European Network of Gynaecological Cancer Advocacy Groups (ENGAGe)
Source: Cancer Med. 2020 Nov 18;10(1):208–19. doi: 10.1002/cam4.3605 (PMC7753798; doi:10.1002/cam4.3605)
Supplement: Supplementary file 1 — Supplementary Material [file CAM4-10-208-s001.docx]

Dear Patients,

In this study, we aimed to evaluate the impression of COVID pandemic over the gynaecological cancer patients and the

patients’ attitudes and experiences upon modifications of their cancer care. Evaluating the depression and anxiety scores

of gynaecological cancer patients during the pandemic are the secondary aims of the study.

Participation in this study is completely voluntary. If you decide not to participate there will not be any negative consequences.

Please be aware that if you decide to participate, you may stop participating at any time and you may decide not to answer

any specific question. There will be no specific question to your private information such as name, surname or identity

number etc. You may fill the survey either by an online survey that your doctors provide to you, or use the hard copy survey

forms given to you. The researcher will maintain the confidentiality of the research records or data.

**SECTION A: DEMOGRAPHIC and CLINICAL INFORMATION**

1. Age:
2. Country:
3. QUESTIONNAIRE NUMBER (PLEASE DO NOT GIVE ANY PERSONAL IDENTITY DATA) :
4. Diagnosis (Ovarian, uterine, cervical cancer, others):
5. Treatment (Type 1, Type 2, Type 3):

(Type 1: Preoperative patients - surgery is planned for a new diagnosis of cancer or recently recurred cancer)

(Type 2: Receiving chemotherapy or radiotherapy, before or after surgery, for primary or recurrent disease)

(Type 3: Not receiving any treatment, only on follow-up)

Do you have any additional diseases (such as diabetes, heart- kidney, liver- or lung disease, increased blood pressure, increased cholesterol levels etc)

A. YES B. NO

If yes – how many different other diseases:

a) 1

b) 2

c) 3 or more

d) None

1. Do you take medication because of a psychological problem or disease?
2. Yes B. No
3. If yes, What Type of Problem……………………….

Which medicine: ……………

How many months have you been taking: ………?

**SECTION B: IMPACT of COVID PANDEMIC ON PATIENTS and THE PATIENTS’ ATTITUDES and EXPERIENCES**

1. PLEASE MARK HOW STRONGLY YOU AGREE OR DISAGREE ACCORDING TO THE FOLLOWING STATEMENTS FROM THE TABLE DURING COVID EPIDEMIC:

|  | Strongly disagree | Disagree | Neither agree nor disagree | Agree | Strongly agree |
| --- | --- | --- | --- | --- | --- |
| 10a. I'm more afraid of cancer compared to COVID. |  |  |  |  |  |
| 10b. I'm more afraid of COVID compared to cancer. |  |  |  |  |  |
| 10c. I think cancer patients have a higher risk of COVID infection. |  |  |  |  |  |
| 10d. I think that chemotherapy suppresses the immune system and creates a predisposition for COVID infection. |  |  |  |  |  |
| 10e. I am afraid of getting COVID infection from the hospital setting while receiving my treatment / follow-up. |  |  |  |  |  |
| 10f. I am concerned about not being able to visit my oncology doctor during the COVID epidemic. |  |  |  |  |  |
| 10g. I am concerned about the progression of my disease if my treatment / follow-up is cancelled/postponed. |  |  |  |  |  |

1. During the COVID pandemic;
2. Due to the fear of COVID infection, I did not go for my check-ups at the hospital.
3. I wanted to go myself, but my doctors cancelled my appointments.
4. It was a joint decision and we postponed our appointment together.
5. My care continued as planned during the COVID pandemic
6. OTHERS:…………………………………………………………………………………………………………………………
7. Are there patients with COVID in the hospital where you are being treated / followed up?
8. Yes, there are B. No, there aren’t C. I don’t know
9. Have you received a COVID test before or during your treatment?
10. Yes B. No
11. Did any of the healthcare professionals giving your medical treatment have COVID infection?
12. Yes B. No C. I don’t know
13. What is the most challenging problem in this period? Please describe ………………………………………………………………………………………………………………………………………………………………………………………………………………………………………………………………………………………………………………………………………………………………………………………………………………………………………………
14. Has your treatment / follow-up been changed due to the COVID pandemic?
15. Yes B. No C. I don’t know
16. If yes, what was done? (multiple responses can be given)
17. I had difficulty reaching my doctor
18. My surgery was delayed
19. My surgery was not delayed, but less radical.
20. My imaging (Tomography, CT, MR, PET, Ultrasound) tests are canceled or disrupted
21. My chemotherapy was delayed
22. My radiotherapy was delayed
23. I received a lower dose of chemotherapy
24. I received a lower dose of radiotherapy
25. My follow-ups are delayed
26. Other (Please describe) ……………………………………………………………………………………

………………………………………………………………………………………………………………………….

1. If your treatment has been postponed, how long has it been postponed:
2. No, it was not postponed.
3. ______ weeks postponed.
4. I don’t know the new time
5. Did you receive any drug as part of a clinical research?
6. Yes B. No C. I don’t know
7. If yes, has the study stopped because of the COVID pandemic and you have no further access to the drug?
8. Yes B. No
9. If yes, it is stopped; would you like to continue in the study?
10. Yes B. No
11. MESSAGE YOU WANT TO SHARE ABOUT COVID PANDEMIA WITH ESGO and ENGAGE or OTHER INTERNATIONAL ORGANIZATIONS AND INSTITUTIONS: ………………………………………………………………………………………………………………………………………………………………………………………………………………………………………………………………………………………………………………………………………………………………………………………………………………………………………………………………………………………………………………………………………………………………………………………………

Please go to the next page for Hospital Anxiety and Depression Scale (HADS).

**SECTION C: EVALUATION of ANXIETY LEVELS IN GYNAECOLOGICAL CANCER PATIENTS**

**Hospital Anxiety and Depression Scale (HADS)**

Tick the box beside the reply that is closest to how you have been feeling in the past week.

Don’t take too long over you replies: your immediate is best.

**23) I feel tense or 'wound up':**

Most of the time

A lot of the time

From time to time, occasionally

Not at all

**24) I still enjoy the things I used to enjoy:**

Definitely as much

Not quite so much

Only a little

Hardly at all

**25) I get a sort of frightened feeling as if something awful is about to happen:**

Very definitely and quite badly

Yes, but not too badly

A little, but it doesn't worry me

Not at all

**26) I can laugh and see the funny side of things:**

As much as I always could

Not quite so much now

Definitely not so much now

Not at all

**27) Worrying thoughts go through my mind:**

A great deal of the time

A lot of the time

From time to time, but not too often

Only occasionally

**28) I feel cheerful:**

Not at all

Not often

Sometimes

Most of the time

**29) I can sit at ease and feel relaxed:**

Definitely

Usually

Not Often

Not at all

**30) I feel as if I am slowed down:**

Nearly all the time

Very often

Sometimes

Not at all

**31) I get a sort of frightened feeling like 'butterflies' in the stomach:**

Not at all

Occasionally

Quite Often

Very Often

**32) I have lost interest in my appearance:**

Definitely

I don't take as much care as I should

I may not take quite as much care

I take just as much care as ever

**33) I feel restless as I have to be on the move:**

Very much indeed

Quite a lot

Not very much

Not at all

**34) I look forward with enjoyment to things:**

As much as I ever did

Rather less than I used to

Definitely less than I used to

Hardly at all

**35) I get sudden feelings of panic:**

Very often indeed

Quite often

Not very often

Not at all

**36) I can enjoy a good book or radio or TV program:**

Often

Sometimes

Not often

Very seldom
